# Plasmonic Nanoparticles for Photothermal Therapy: Benchmarking of Photothermal Properties and Modeling of Heating at Depth in Human Tissues

**DOI:** 10.1021/acs.jpcc.4c06381

**Published:** 2025-01-09

**Authors:** William H. Skinner, Marzieh Salimi, Laura Moran, Ioana Blein-Dezayes, Megha Mehta, Sara Mosca, Alexandra-Geanina Vaideanu, Benjamin Gardner, Francesca Palombo, Andreas G. Schätzlein, Pavel Matousek, Tim Harries, Nick Stone

**Affiliations:** † Department of Physics and Astronomy, University of Exeter, Exeter EX4 4QL, U.K.; ‡ Central Laser Facility, STFC Rutherford Appleton Laboratory, Oxford OX11 0QX, U.K.; § School of Pharmacy, University College London, London WC1N 1AX, U.K.

## Abstract

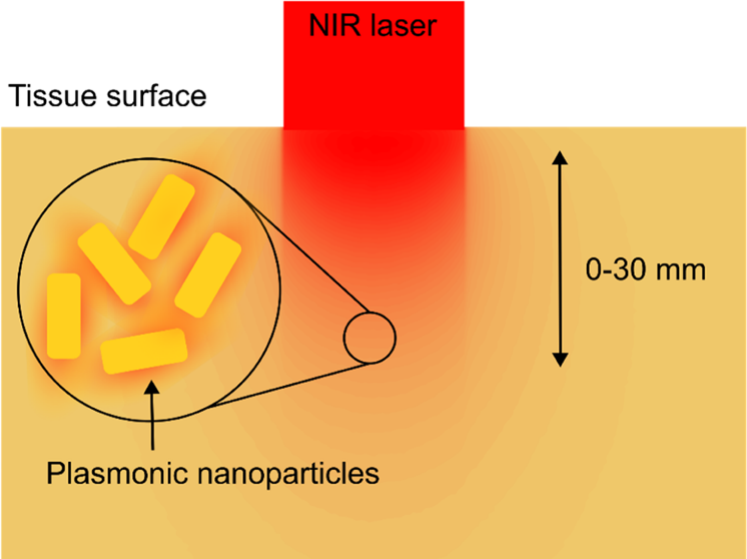

Many different types of nanoparticles have been developed for photothermal therapy (PTT), but directly comparing their efficacy as heaters and determining how they will perform when localized at depth in tissue remains complex. To choose the optimal nanoparticle for a desired hyperthermic therapy, it is vital to understand how efficiently different nanoparticles extinguish laser light and convert that energy to heat. In this paper, we apply photothermal mass conversion efficiency (η*_m_*) as a metric to compare nanoparticles of different shapes, sizes, and conversion efficiencies. We selected silica-gold nanoshells (AuNShells), gold nanorods (AuNRs), and gold nanostars (AuNStars) as three archetypal nanoparticles for PTT and measured the η*_m_* of each to demonstrate the importance of considering both photothermal efficiency and extinction cross section when comparing nanoparticles. By utilizing a Monte Carlo model, we further applied η*_m_* to model how AuNRs performed when located at tissue depths of 0–30 mm by simulating the depth penetration of near-infrared (NIR) laser light. These results show how nanoparticle concentration, laser power, and tissue depth influence the ramp time to a hyperthermic temperature of 43 °C. The methodology outlined in this paper creates a framework to benchmark the heating efficacy of different nanoparticle types and a means of estimating the feasibility of nanoparticle-mediated PTT at depth in the NIR window. These are key considerations when predicting the potential clinical impact in the early stages of nanoparticle design.

## Introduction

Photothermal therapy (PTT) combines light-absorbing photothermal agents and deep tissue penetrating NIR radiation to induce local hyperthermia at the site of solid tumors, resulting in cell death and tumor shrinkage. Successful treatment with PTT depends on two factors: the temperature increase at the tumor site and the duration of the heating.^1−3^

Gold nanoparticles are promising photothermal agents because of their strong absorption cross sections and biocompatibility.^4^ NIR-absorbing gold-silica nanoshells have reached clinical trials for treating prostate tumors, and many other nanoparticles for PTT continue to be developed.^5−7^ In parallel, advances in NIR spectroscopy are beginning to enable temperature measurements from nanoparticles at depth during PTT.^8^ Most nanoparticles developed for photothermal therapy absorb strongly in the first NIR tissue window (∼650–950 nm); where light has improved tissue penetration.^9^ Working within the first NIR tissue window could enable the optical excitation of nanoparticles in nonsuperficial tumors and reduce off-target heating in healthy tissue when nanoparticles are sufficiently localized to the tumor.^10^ For accessible tumors, NPs may be injected directly in the tumor; this typically leads to nonuniform distributions of photothermal agents which when activated, act as hotspots, reaching very high temperatures compared to the rest of the tumor. In contrast, systemic administration may lead to uniform accumulation at a desired site. However, the efficiency of delivering intravenously injected nanoparticles to tumors remains low, with a review paper calculating a median of 0.7% of administered nanoparticle doses delivered to solid tumors.^11^ Therefore, an efficient transducer of light-to-heat energy is a key attribute of PTT NPs. Furthermore, a thorough understanding of how nanoparticle type, laser power, concentration at the tumor site, and tumor depth influence heating is key to predicting the potential clinical impact of PTT nanoparticles.

A vast array of nanophotothermal agents have been developed for PTT, from nanoparticles such as nanoshells,^12^ nanorods^13^ and nanostars,^14^ to more complex nanoassemblies such as “core–satellite” nanoparticles^15^ and “core-multitentacle” nanoassemblies.^16^ These nanoparticles and nanoassemblies are designed to have strong surface plasmon resonance (SPR) in the first NIR tissue window. This creates a strong interaction between the oscillating electric field of the laser light and the surface free electrons of the metallic nanoparticles. As the surface electrons’ collective and coherent oscillation decays energy is dissipated as heat to the surroundings or reradiated as scattered photons.^10,17^ Nanoparticles are typically characterized by the sum of their scattering (μ_scat_) and absorbance (μ_abs_) properties via the extinction coefficient (μ) (eq 1)
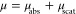
1Only absorbed light, μ_abs_, contributes to heating. The experimentally determined fraction of extinguished light contributing to heating is the photothermal conversion efficiency (η) and is calculated using energy balance equations.^18−20^ But the mass of NPs required to generate local heating is also important when comparing photothermal agents, and this is not captured by η.

Researchers have tackled this characterization and ranking challenge by evaluating nanoparticle heating as a function of nanoparticle absorption cross-section and nanoparticle mass concentrations. Cole et al. examined the photothermal conversion efficiency of gold nanorods and silica-gold nanoshells.^21^ They showed that nanorods had around double the photothermal conversion efficiency of nanoshells, but on a single-particle basis, nanoshells converted more light energy to heat because of their much larger extinction cross-section. Paściak et al. developed ‘external heating efficiency’ as a metric for the quantitative ranking of nanoheaters.^19^ They multiplied the photothermal conversion efficiency of different nanoparticles by their mass absorption coefficient to account for how efficiently different nanoparticles coupled to the laser light. Using external heating efficiency, they were able to quantitatively compare heating from gold nanorods and copper sulfide nanoparticles, among other types of strongly absorbing nanoparticles. However, as the authors note, their approach only applies to highly absorbing nanoparticles, and many nanoparticles and nanostructures developed for PTT have a significant scattering contribution.^21^ Balfourier et al. characterized suspensions of aggregated gold nanoparticles using a “specific absorption rate” that captures the initial slope of the temperature increase when limited thermal exchange occurs between the sample volume and the surrounding atmosphere and normalized it to the gold mass concentration in the suspension. This produced a metric to compare the heating efficiency of 30 nm gold nanoparticle aggregates of different fractal dimensions.^22^

In this paper, we directly compare nanoparticles of different shapes and sizes to examine differences in their optical properties and normalize them to experimental conditions such as laser power, beam width, and sample volume. We call this metric the photothermal mass conversion efficiency (η_*m*_) and believe it will aid the characterization and ranking of nanoparticles developed for PTT by normalizing heating efficiency to the mass of the photothermal agent. We selected gold-silica nanoshells, nanorods, and nanostars with a surface plasmon resonance in the NIR biological window and characterized their absorption and scattering properties by standard methods. We then calculated η_*m*_ for each nanoparticle type and explored the ranking of nanoheaters with this metric. Finally, we set out to provide a framework to link the illuminating photon fluence and the nanoparticle η_*m*_ to its practical PTT performance when buried within tissue, thus demonstrating the practical limitations of the PTT approach at depth using different parameters.

## Methods

### Materials

Gold nanorods and gold-silica nanoshells were purchased from NanoComposix (GRCN800 and GSCR150). Gold nanostars were purchased from Nanopartz (A1S-780). Cuvette stir bars were purchased from Sigma-Aldrich (Z363545). K-type thermocouples and Pico TC-08 Thermocouple Data Logger from Pico Technology. USB2000-vis-NIR-ES spectrometer from Ocean Optics, FL, USA.

### Nanoparticle Characterization

Gold nanorods (AuNRs) (length = 33.2 nm, width = 9.1 nm), gold-silica nanoshells (AuNShell) (*r*_1_ = 59.9 nm, *r*_2_ = 76.4 nm), and gold nanostars (AuNStar) (total diameter 100 nm) were selected because they have a surface plasmon resonance at ∼800 nm, which makes it appropriate for a comparative analysis of PTT performances. All nanoparticles were stabilized in citrate buffer. The suspensions of each nanoparticle type were diluted in ultrapure water to an extinction of 0.1 at 808 nm (laser wavelength) for all experiments. We determined the mass concentration of gold for each colloid by inductively coupled plasma mass spectrometry (ICP-MS) (full protocol in Supporting Information). To directly compare the heating achieved per mass of particles, we estimated the mass contribution of the silica core of AuNShells from the mass of gold measured with ICP-MS. From the *r*_1_ and *r*_2_ of AuNShells, 48% of the volume of an AuNShell particle is silica; the density of silica is 2.65 g/cm^3^, and that of gold is 19.32 g/cm^3^. Hence, the mass concentration of gold measured by ICP-MS for AuNPs was increased by 12.7% to account for the contribution of the silica core to the total mass of nanoparticles. Transmission electron microscopy (TEM) images were collected on a JEOL 1400 JEM. The mass extinction coefficient (ε_*m*λ_) for each nanoparticle type at 808 nm was determined using Beer–Lambert law in mass form (eq 2).^19^ Where ε_λ_ is the extinction at 808 nm, *C*_NPs_ is the mass concentration (mg/mL) and *L* is the optical path length (cm).
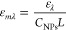
2

### Photothermal Conversion Efficiency

The photothermal conversion efficiency (η) was calculated for each nanoparticle type using Roper et al.’s method.^20^ Nanoparticle suspensions were placed in quartz cuvettes and heated with an 808 nm laser for 30 min to reach equilibrium and then allowed to cool to ambient temperature. At equilibrium, the power entering the system from the laser is equal to the power lost to the surroundings and the η can be calculated from eq 3 (a modified version of the equation introduced by Roper et al. see Supporting Information 1.1)
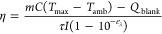
3where *m* and *C* are the mass (2 g) and heat capacity (4.182 J·g^–1^·K^–1^) of the nanoparticle suspension. *T*_max_ is the equilibrium temperature, *T*_amb_ is the ambient temperature, *Q*_blank_ is energy dissipated in a cell containing 2 mL of distilled water (J), *I* is the power of the laser beam after attenuation by a blank quartz cuvette filled with water (1 W) and ε_λ_ is the extinction of the suspension at 808 nm. τ is the time constant calculated from the cooling data using eq 4, a solution to Newton’s cooling law, where *T* is the temperature of the nanoparticle suspension and *t* is the time since the laser was turned off.
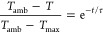
4All samples were equilibrated to room temperature before laser-induced heating. Stirring was performed with a stir bar designed for rapid horizontal and vertical mixing in a cuvette and temperature was measured with K-type thermocouples and a Pico TC-08 Thermocouple Data Logger. The scattered light was collected at 90° to the laser path and measured using a fiber-coupled Ocean Optics UV–vis-NIR spectrometer. An image of the experimental setup is presented in the Supporting Information (Figure S1).

### Photothermal Mass Conversion Efficiency

To calculate the heating efficiency of nanoparticles as a function of mass we use photothermal mass conversion efficiency (η_m_), which we define as the light absorbed per μg of nanoparticle in the laser path according to eq 5.
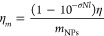
5where σ is the extinction cross-section of the particles [m^2^], *N* is the number density of particles [m^–3^], and *l* is the path length through the sample [m]. *m*_NPs_ is the mass of nanoparticles in the path length of the laser (excluding the beam broadening effect of any light scattering) and was calculated for a beam area of 8.1 mm^2^ (fwhm measured with a Thorlabs beam profiler BC106N-VIS/M) and a cuvette width of 10 mm. By substituting (3) into (5) we obtain
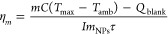
6At short laser exposure times and low temperature elevations, heat transfer between the cuvette and the ambient surroundings is approximated to zero. Under these conditions, the energy input of the laser results in heating above the ambient temperature. Therefore, under the principle of energy balance:
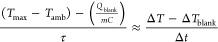
7where Δ*T* is the temperature increase in the nanoparticle suspension at Δ*t =* 60 s of laser heating and Δ*T*_blank_ the heating of the blank cuvette (with 2 mL volume of distilled water). Combing (6) and (7), we obtain eq 8 which enables the calculation of η_*m*_ without prior knowledge of η and with significantly less experimental time devoted to heating.
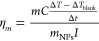
8*I* is the power of the laser after attenuation by a blank quartz cuvette filled with water, for photothermal mass conversion efficiency experiments this was set to 500 mW.

### Modeling Light Penetration through Tissue

Monte Carlo codes simulate light transfer by dividing the radiation field into a large number of indivisible photon packets that propagate through the computational domain following a random walk dictated by the absorption and scattering coefficients of the underlying medium. By running a sufficient number of photon packets, a statistical picture of light transport through a medium can be built up. We used a custom-written Monte Carlo model based on numerical algorithms from the astrophysical code TORUS.^23,24^ This flexible model is called arctk, and is detailed in Wordingham’s et. al’s work.^25^ An early version of the codebase arctk has also been used to simulate skin cancer PTT at shallow depths.^26^

We simulated a breast tissue volume of 8 cm × 8 cm × 5 cm which was given the optical properties μ_s_’ = 11.7 cm^–1^ and μ_a_ = 0.035 cm^–1^ at 808 nm.^27^ Into this volume, a 1 W Gaussian laser beam of 808 nm photons was modeled with a beam diameter of 10 mm, and the energy density at each voxel in the breast tissue volume was obtained. Each photon packet was directed at a normal vector to the front face to model a collimated laser beam. This simulated volume of energy density was then scaled to explore power densities of 1, 3, and 5 W/cm^2^ at the tissue surface. The energy density throughout the tissue was converted into an equivalent power density in each voxel, enabling the heating achievable with nanoparticles in a particular voxel to be calculated. It should be noted that this simulation is an idealized model, where NPs are only present at the target voxel and there is assumed to be no absorbance from other NPs between the surface and the target voxel. The value of the simulation lies in demonstrating the maximum possible NP localized heating rate at depth with specific laser illumination.

To estimate the heating rate from a single voxel containing nanoparticles, we multiplied the power density in the voxel by the mass of nanoparticles present in the voxel and the corresponding photothermal mass conversion efficiency (eq 9).
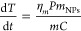
9where  (K/s) is the peak heating rate assuming no thermal equilibrium with surroundings, η*_m_* is the photothermal mass conversion efficiency (μg^–1^), *P* is power (W) reaching a voxel at the surface or 10–30 mm below the surface of the tissue model, *m*_NPs_ is the mass of the nanoparticles in the voxel (μg), *m* is the mass of tissue in the voxel (7.47 μg) and *C* is the specific heat capacity of water (4.186 J·g^–1^·K^–1^). The heating ramp time to reach 43 °C was then calculated by dividing the difference between body temperature (37 °C) and 43 °C by .

## Results and Discussion

### Nanoparticle Optical Characterization

**Figure 1:**
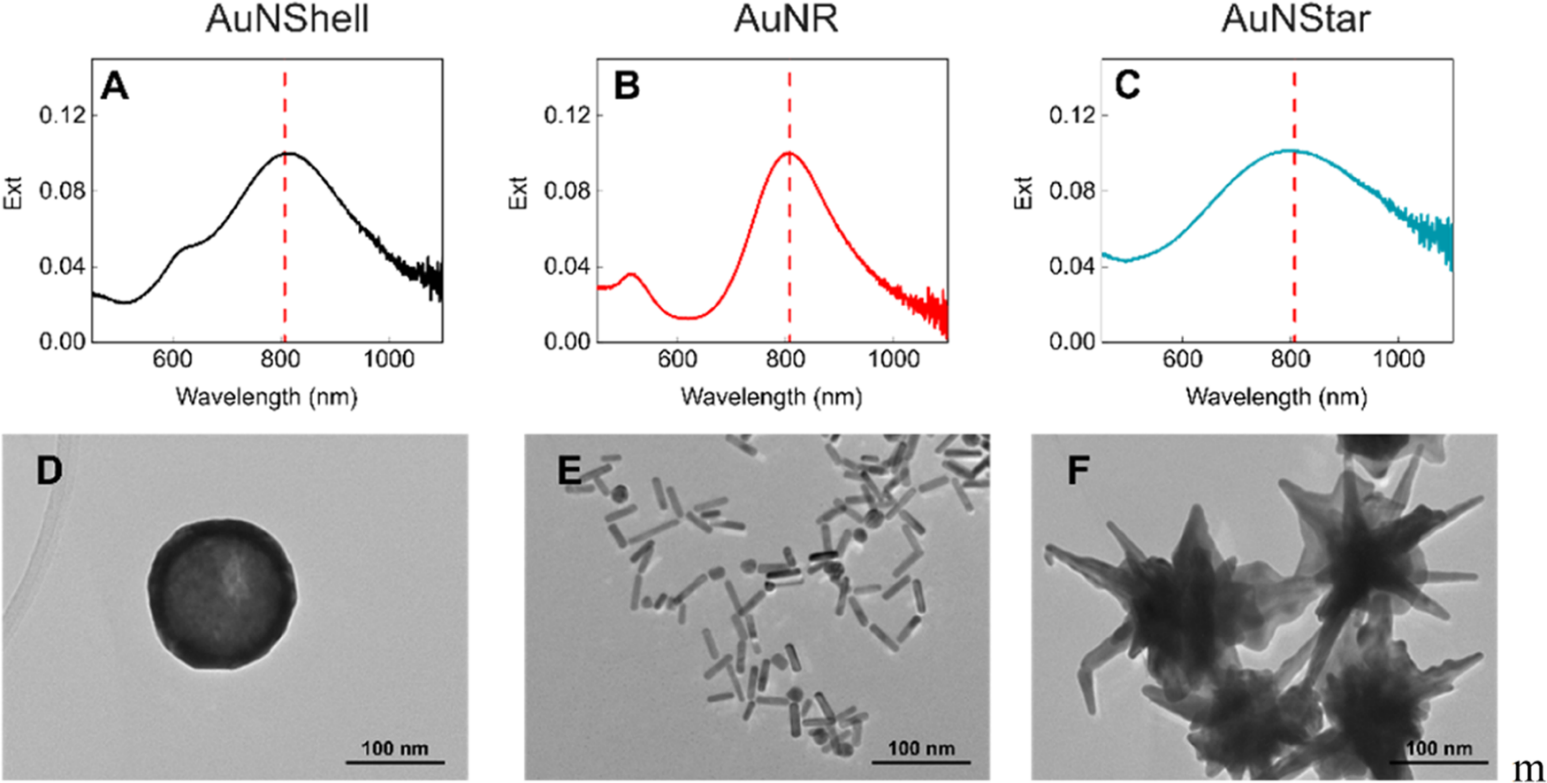
UV–vis extinction spectra of (A) AuNShells, (B) AuNRs, and (C) AuNStars. The red dashed line indicates the laser wavelength used for PTT (808 nm). TEM image of (D) AuNShell, (E) AuNRs, and (F) AuNStars. All TEM images were collected at the same magnification.

We selected nanoshells (AuNShells), nanorods (AuNRs) and nanostars (AuNStars) for our study because of their widespread application to PTT research. All three nanoparticle types had a peak surface plasmon resonance at ∼800 nm (Figure 1), but we expected the differences in size and shape of these nanoparticles to lead to significant differences in their absorption and scattering properties and hence their performance as nanoheaters for PTT.

AuNShells (Figure 1A,D) have a spherical silica core surrounded by a gold shell. Their surface plasmon position is dependent on both the core–shell ratio (*r*_1_/*r*_2_) and the total radius.^28^ AuNRs (Figure 1B,E) have two resonances. The weaker transverse mode occurs at ∼520 nm, and the stronger longitudinal mode occurs at ∼800 nm. The position of the longitudinal mode is dependent on the aspect ratio of the nanorods.^18^ AuNStars (Figure 1C,F) have a central spherical core with spikes extending outward. The length and width of these spikes influence the position of the surface plasmon resonance.^29^

Despite all three nanoparticles exhibiting an SPR at ∼800 nm, TEM images indicated that the size of individual nanoparticles varies drastically, as do the concentrations of each nanoparticle in a solution exhibiting an extinction of 0.1 at 808 nm (79% transmission) (Table S1). All heating experiments were carried out on nanoparticle suspensions with an extinction at 808 nm of 0.1 (Figure 1 A–C). This ensured that the attenuation of laser light through the sample was consistent for each nanoparticle and allowed the direct comparison of heating and scattering properties. UV–vis spectra collected before and after heating confirmed the nanoparticles had not aggregated during the experiment (Figure S2). The temperature increase from a nanoparticle-free blank cuvette containing water led to a minimal temperature increase because no appreciable absorbance occurs at 808 nm for quartz or water (Figure S3).

**Figure 2:**
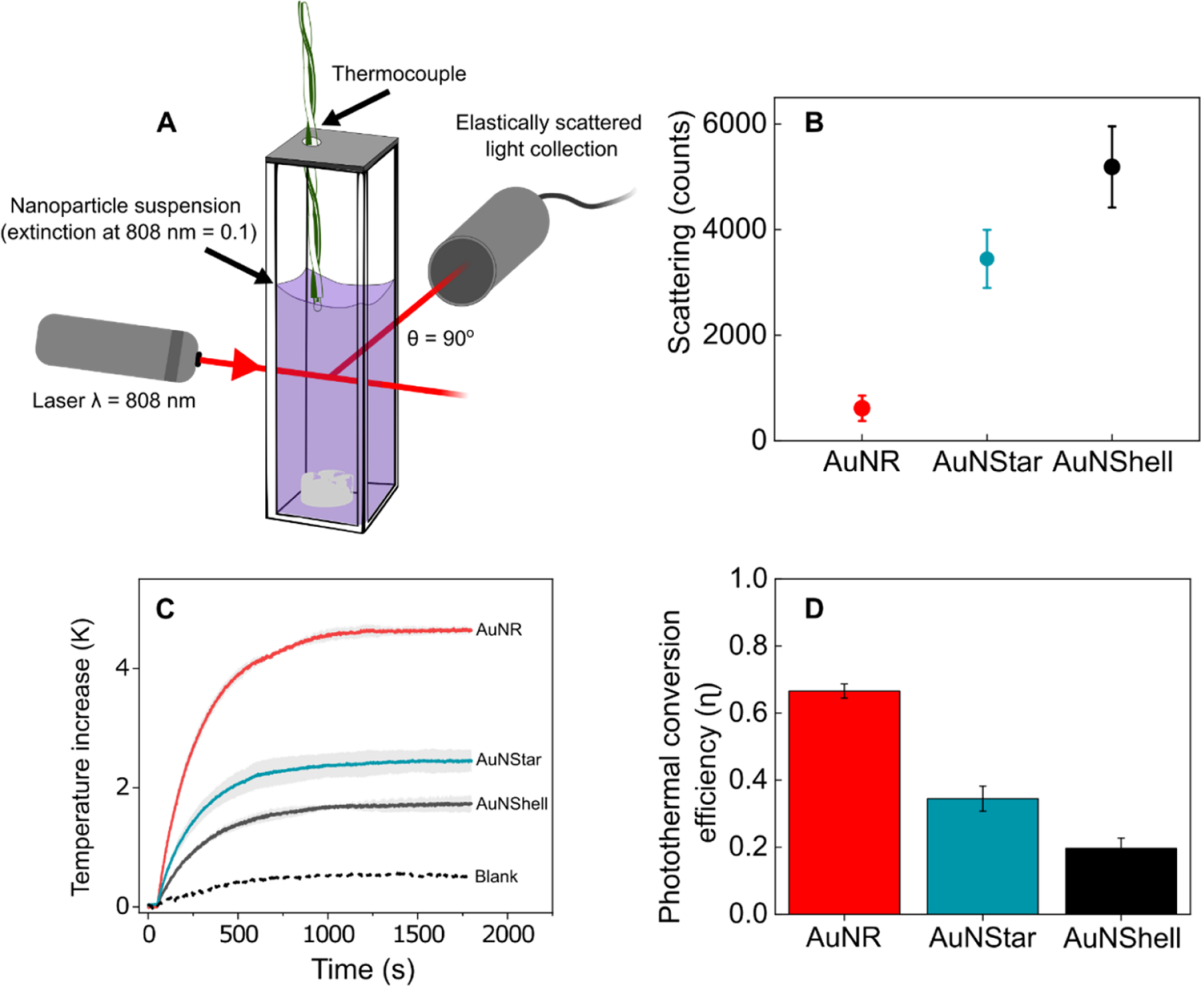
(A) Schematic of the experimental setup, all three suspensions were diluted to an extinction value of 0.1 at 808 nm. (B) The intensity of light scattered by AuNShells, AuNRs, and AuNStars during 10 ms 808 nm laser exposure at 500 mW power. (C) The temperature increase of the nanoparticle suspensions and blank relative to ambient temperature upon exposure to laser at 1 W. (D) Photothermal conversion efficiency of the nanoparticles (SD of 3 technical repeats).

As outlined above, we used Roper et al.’s method to calculate the photothermal conversion efficiency of each nanoparticle suspension.^20^ A schematic of the experimental setup is presented in Figure 2A, and an image is included in Figure S1. Because each nanoparticle suspension has an extinction value of 0.1 at 808 nm, the heating curves can be directly compared, and it is evident that nanorods have a higher photothermal conversion efficiency with an equilibrium temperature increase of 4.6 °C, almost double that of AuNStars (2.5 °C) and AuNShells (1.7 °C) (Figure 2C). Using eq 3, nanorods were found to have the highest photothermal conversion efficiency, with η = 0.66, while nanostars had η = 0.34, and nanoshells were the lowest with η = 0.20 (Figures 2D and S4). These values are consistent with those previously reported for similar-sized nanorods and nanoshells.^21^ Nanorods often have larger η because their elongated structure increases the surface-to-volume ratio, which increases the percentage of nanoparticle volume contributing to resistive heating from surface plasmon oscillations.^30^ While heat power density can be nonuniform in nanoparticles such as AuNR, the temperature distribution at equilibrium within such particles is near uniform due to the high thermal conductivity of gold compared to the aqueous surroundings. Furthermore, at the laser powers and concentrations used in our work, heating is a collective effect of many nanoparticles creating a uniform temperature distribution, despite the nanoscale of the heat source, allowing accurate measurements of η from bulk thermocouple measurements.^31^

The trend in photothermal conversion efficiency observed in Figure 2D was also confirmed by the magnitude of light scattered by each nanoparticle type during exposure to the 808 nm laser, which showed the reverse of the photothermal conversion efficiency trend (Figure 2B). It should be noted that these photothermal conversion efficiency values are not general to all AuNRs, AuNStars and AuNShells, but rather reflect the specific dimensions and geometries of the nanoparticles used in this study. For example, increasing the volume of AuNRs while maintaining their aspect ratio has been shown to decrease their photothermal conversion efficiency.^18^

### Photothermal Mass Conversion Efficiency

Photothermal conversion efficiency captures the fraction of light converted into heat but not the efficiency with which incoming light is extinguished. For example, previous reports have noted that nanorods can generate more heat per μg than gold nanoshells.^32^ Furthermore, gold nanocages (another nanoparticle geometry) have been shown to generate twice as much heat per μg than nanorods,^33^ though other studies have found no such superior properties of nanocages.^34^ Given the interest in quantifying the heating efficiency of nanoparticles in terms of mass, we propose photothermal mass conversion efficiency (η_*m*_) (eq 8) as a means of capturing two metrics essential to the efficiency of nanoparticles for PTT: the photothermal conversion efficiency and the mass extinction efficiency. Maximizing the heating efficiency per μg of nanoparticle is vital because, as mentioned above, nanoparticle delivery to tumors remains low. Furthermore, photothermal characterization based on nanoparticle mass is more intuitive for researchers focusing on the required nanoparticle delivery to tumors where nanoparticle accumulation is measured with ICP-MS yielding a mass of gold per tissue mass. This photothermal mass conversion efficiency could aid in selecting appropriate nanoparticles for treatment when comparing different geometries and sizes of nanoparticles.

**Figure 3:**
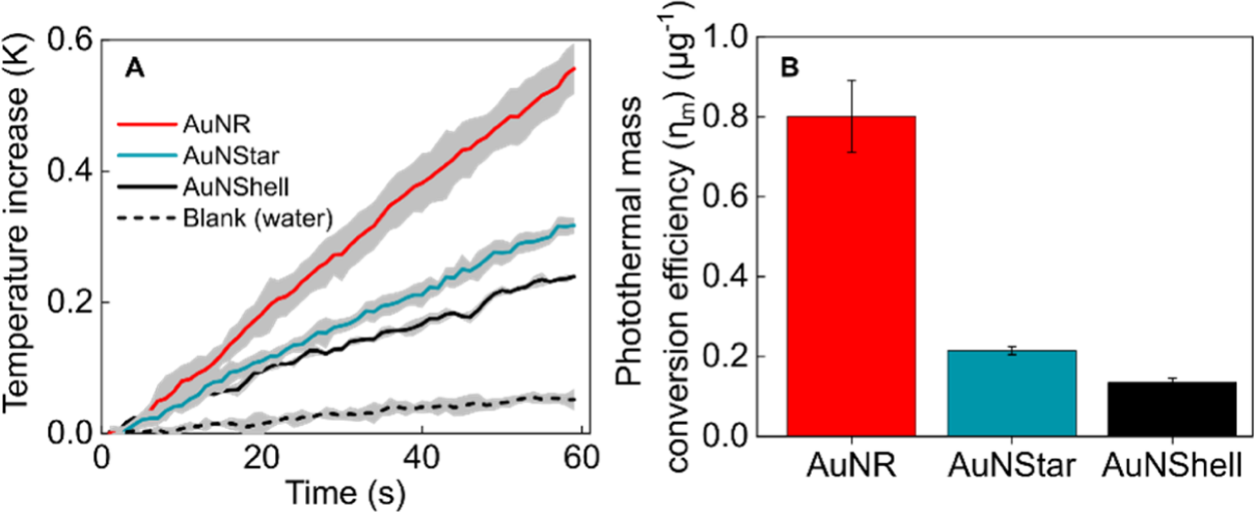
(A) Relative temperature increase of the three nanoparticle suspensions and a water blank during the first 60 s of laser irradiation (808 nm, 500 mW). The solid line is the mean and shading is the SD of 3 technical repeats. (B) Photothermal mass conversion efficiency of AuNShells, AuNRs, and AuNStars. Error bars indicate the SD of 3 technical repeats and SD in gold mass concentrations.

To calculate photothermal η_m_ for each nanoparticle type, we measured the slope of the temperature–time trace in the first 60 s following irradiation with 500 mW of 808 nm laser light (Figure 3A); under these conditions, thermal equilibrium between the sample volume and ambient surroundings is minimized, and heat transfer is approximated to zero. To ensure we only measured the heating of the NPs and to account for direct laser heating of the water and quartz cuvette in our calculations, we first calibrated the temperature increase under the same parameters within a water-only sample, which showed a much lower temperature increase. Using eq 8, we calculated η_m_ for each nanoparticle type (Figure 3B). The trend observed in photothermal conversion efficiency (Figure 2D) remained the same but the magnitude of the differences between the nanoheaters was altered because the mass extinction coefficient, which can be determined from the Beer–Lambert law, is different for each nanoparticle type (Table 1).

**Table 1:** Mass Extinction Coefficients (ε_*m*λ_) of AuNShells, AuNRs, and AuNStars

nanoparticle type	ε_*m*λ_ (cm^2^/mg)
AuNshells	31.7 ± 0.8
AuNRs	46.1 ± 8.4
AuNstars	24.7 ± 0.6

In Figure 2D, the photothermal conversion efficiency of AuNStars was 48% lower than that of AuNRs. When the larger mass extinction coefficient of AuNRs is accounted for, using η_m_, the difference increases, and AuNStars are 73% less effective than AuNRs. Likewise, the difference between the heating performance of AuNStars and AuNShells decreased when η_m_ was measured (Figure 3B) because AuNShells possess a larger mass extinction coefficient at 808 nm than AuNStars (Table 1). Intuitively, nanoparticles with a plasmon mode out of resonance with the laser will yield lower η_m_ values than nanoparticles of a similar geometry in resonance with the laser (Figure S5).

To validate our derivation of eq 8 and our η_m_ measurements, we used the results from Figure 3B to calculate photothermal conversion efficiency (η) using eq 5. We compared these results to η measured in Figure 2D with Roper et al.’s method. The η calculated with both methods were in close agreement, validating our derivation and experimental method for η_m_ measurement (Figure S6).

AuNRs had the largest mass extinction coefficient, combined with the largest η. This set AuNRs apart from the AuNShells and AuNStars in terms of heating achievable per μg of gold. The advantage of η_m_ measurements is to capture the mass extinction coefficient and the photothermal conversion efficiency in a single metric, which allows the direct comparison of nanoparticles of different geometries with varying scattering and absorbance properties. This is important to consider in situations where only a fraction of the laser light is extinguished by the nanoheaters, for example, in tissues containing low concentrations of nanoparticles.

### Laser Tissue Penetration

**Figure 4:**
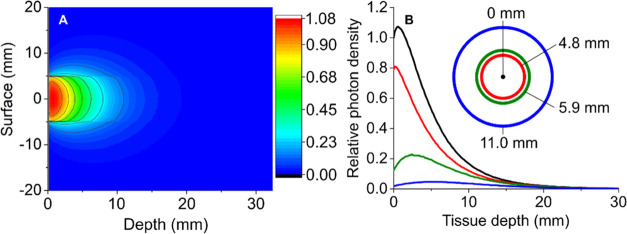
(A) Heat map of simulated photon penetration into breast tissue. Photon density is presented as a fraction of the dose delivered to the tissue surface. (B) Relative photon density at depths of 0–30 mm for distances laterally translated from the optical axis (center of the laser spot) by 0, 4.8, 5.9, and 11 mm.

To date, nanoparticle-mediated PTT has reached clinical trials for the treatment of prostate cancer and has been suggested as a treatment for other solid tumors.^5,35,36^ To this end, it is vital to understand the penetration of 808 nm light into tissue to determine maximum treatment depths for large tumor masses or when nonsuperficial tumors are targeted. Biological tissue significantly attenuates laser light, mostly through elastic scattering, even in the NIR window. Here we explored the fraction of the laser light that reaches depths of 0–30 mm in breast tissue with Monte Carlo simulations (Figure 4).

The simulated tissue volume was chosen to represent breast tissue, with a depth of 5 cm, to replicate the conditions of a mammogram. Breast cancer is one of the potential targets of PTT so these simulation conditions were deemed appropriate. The laser beam area was modeled as a circle with a diameter of 10 mm. The relative energy density at depths of 0–30 mm at various distances from the beam center is presented in Figure 4.

The maximum photon density is achieved at the center of the 10 mm diameter beam and 1–3 mm below the surface (Figure 4). The photon density at this point is higher than the photon density at the surface, as a large fraction of surface scattered photons are lost, while at the near-surface, there is a point where the backscattered photons are mostly retained in the tissue, rather than lost. The photon density then rapidly decreases, primarily due to scattering losses away from the optical axis (with some absorption), reaching 17% of the maximum at 10 mm, 2% at 20 mm and 0.34% at 30 mm. Scattering within the tissue also broadens the beam and significant photon densities occur up to 5 mm away from the edge of the laser spot, illustrating the importance of effective targeting/localization of nanoparticles in the tumor lesion to prevent off-target heating.

Tissue damage induced by hyperthermia depends on the magnitude of the temperature increase and the duration of heating. At temperatures between 43–57 °C, a 1 °C increase in temperature requires approximately half the time to deliver the same level of thermal damage.^3^ For hyperthermia treatments, the thermal isoeffect dose captures the thermal damage to tumor tissues induced by treatments with different temperatures and times as an equivalent number of minutes at 43 °C. We explored how the photothermal mass conversion efficiency (η_m_) of nanoparticles combined with simulations of photon penetration through tissue could be used to approximate the minimum time required for nanoheaters to reach a temperature threshold of 43 °C at depths of 0–30 mm in tissue, excluding effects of background heating from surrounding tissue.

Using an idealized model, we modeled the application of 1–5 W/cm^2^ of laser power to the surface of our tissue and estimated the minimum time required to reach a hyperthermic temperature of 43 °C in a single voxel containing a range of AuNR concentrations (Tables 2, S2, and S3). A range of 0.005–0.020 mg/g of AuNRs was chosen to reflect the gold concentrations of nanoparticles that accumulated in human prostate tumors via the enhanced permeability and retention effect following intravenous injection in a recent study.^5^ We also used laser irradiances similar to and below those used in this clinical study. We modeled heating at different tissue depths assuming no thermal conduction with surrounding tissue and that all other voxels in the model contain no gold, i.e., perfect nanoparticle targeting to the tumor/voxel with none in surrounding healthy tissue.

**Table 2:**
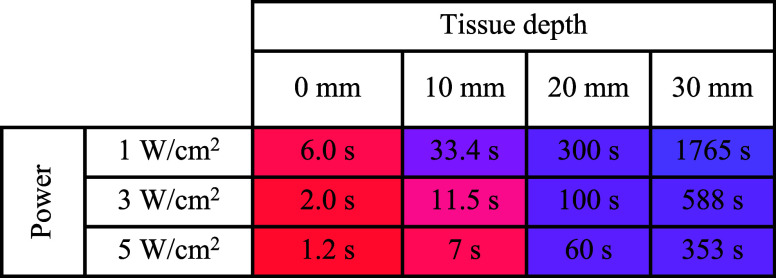
Minimum Time (s) Required for a Voxel Containing 0.01 mg/g of AuNRs to Reach a Hyperthermic Temperature of 43 °C at Different Depths within Breast Tissue

The time constraints of PTT, and hence the maximum depth and minimum nanoparticle concentration required, depend on the treatment goal e.g., thermal ablation, or extended hyperthermia. For a concentration of 0.01 mg/mL, we estimate our target temperature of 43 °C would be reached within 6 s at the surface with the application of 1 W/cm^2^ but would take ∼30 min at a depth of 30 mm, making such a treatment option likely to be nonviable. Increasing laser power to 3 and 5 W/cm^2^ could make treatments at depths 20 mm achievable within a reasonable time frame (<2 min).

Our model outlines the best-case scenario achievable at depth with an 808 nm laser and enables the limits of the approach to be identified. Clearly, increasing nanoparticle concentration increases the depth at which sufficient heating is achieved. However, as previous work has shown, laser attenuation limits depth penetration at extremely high nanoparticle concentrations, leading to a maximum temperature threshold that cannot be increased by increasing nanoparticle concentration.^37^ For heating at depth without insertion of optical fibers, i.e., through “off-target” healthy tissue, it is important to consider that high laser powers alone may result in significant tissue heating, and increased nanoparticle concentrations may be a better approach to achieving the target temperature elevation. To design an effective treatment with our modeling approach, a factor may need to be applied to ensure a greater number of NPs or light is applied based on practical testing of the approach. However, this work demonstrates how a relatively simple model of light penetration through human tissue and empirically determined nanoparticle photothermal properties can be applied to ascertain an approximation of heating rate at depth during PTT.

## Conclusions

In summary, we applied photothermal mass conversion efficiency (η_m_) as an empirical metric to compare the heating efficiency of nanoparticles as a function of nanoparticle mass. This approach enabled the benchmarking of 3 nanoparticle types with a range of scattering and absorbance properties. Our results demonstrate how the most effective nanoparticle heaters have both a high photothermal conversion efficiency and a large mass extinction coefficient, and we captured this in a single metric, η_m_. We then pushed the application of η_m_ further by simulating the penetration of 808 nm laser light through 0–30 mm of human tissue and estimating the heating rate achievable at depth with varying nanoparticle concentrations. This work set out the relationship between nanoparticle concentration, tissue depth, and laser power, providing a simple model which may reduce the requirement for insertion of fiber optic illuminators within the target organ. Further work should develop the idealized model to consider voxel heat loss due to vascularization and the effect of NP distributions within a tumor on the light/heating reaching the “dark side of the tumor”. This would enable treatments to consider the use of multiple external illumination orientations in a similar approach to photon beam radiotherapy.

## Supporting Information Available

ICP-MS protocol and mass concentration of Au in each colloid; additional temperature ramp times for different AuNR concentrations at depth, and optical setup used to collect data (PDF)
