# Supplementary material for: Plasmonic Nanoparticles for Photothermal Therapy: Benchmarking of Photothermal Properties and Modeling of Heating at Depth in Human Tissues
Source: J Phys Chem C Nanomater Interfaces. 2025 Jan 9;129(3):1864–72. doi: 10.1021/acs.jpcc.4c06381 (PMC11770747; doi:10.1021/acs.jpcc.4c06381)
Supplement: Supplementary file 1 — jp4c06381_si_001.pdf [file jp4c06381_si_001.pdf]

SUPPORTING INFORMATION

Plasmonic Nanoparticles for Photothermal Therapy: Benchmarking of Photothermal Properties and Modeling of Heating at Depth in Human Tissues

William H. Skinner,[1‖] Marzieh Salimi,[1†‖] Laura Moran,[1] Ioana Blein-Dezayes,[1] Megha Mehta,[1] Sara Mosca,[2] Alexandra-Geanina Vaideanu,[3] Benjamin Gardner,[1] Francesca Palombo,[1] Andreas G. Schätzlein,[3] Pavel Matousek,[2] Tim Harries,[1] Nick Stone*[1]

[1]Department of Physics and Astronomy, University of Exeter, Exeter EX4 4QL, UK
[2]Central Laser Facility, STFC Rutherford Appleton Laboratory, Oxford OX11 0QX, UK

[3]School of Pharmacy, University College London, London, WC1N 1AX, UK

TABLE OF CONTENTS

Section 1: Methods

Section 1.1: ICP-MS protocol

Section 1.2: photothermal conversion efficiency equation

Section 2: Figures and Tables

Table S1. Mass concentration of Au in suspension of nanoparticles with ε_{808}=0.1

Table S2. Time (seconds) required for a voxel containing 0.005 mg/g of AuNRs to reach a hyperthermic temperature of 43°C at different depths within breast tissue.

Table S3. Time (seconds) required for a voxel containing 0.02 mg/g of AuNRs to reach a hyperthermic temperature of 43 °C at different depths within breast tissue.

Figure S1. Experimental set-up for PTT experiments.

Figure S2. UV-Vis spectra collected before and after irradiating samples for 30 minutes with 1 W of laser power.

Figure S3. UV-Vis spectrum of the blank quartz cuvette containing water.

Figure S4. Representative heating curves used to calculate photothermal conversion efficiency (η) for AuNRs, AuNShells and AuNStars.

Figure S5. Photothermal mass conversion efficiency (η_m) of off-resonance gold nanorods ($AuNR_{746}$) and comparison with on-resonance nanoparticles ($AuNR_{808}$, AuNShell and AuNStar).

Figure S6. A comparison of photothermal conversion efficiency (η) calculated using Roper *et al*.'s method and calculated from η_m

Section 1: Methods

Section 1.1: ICP-MS protocol

Colloidal samples were accurately weighed (10 mg) and digested in aqua regia as follows: samples were allowed to thaw and reach RT, then 800µL of 37% HCl v/v (SpA grade, ROMIL, UK) and 200µL of 69% HNO3 (SpA grade, ROMIL, UK) were added to each vial and samples were allowed to digest uncapped, overnight, in the fume hood. Then, samples were transferred to clean borosilicate tubes and heated on a heating block at 80 ºC overnight, until the solution turned light yellow. Samples were allowed to cool down to RT, were diluted and accurately weighed, to approximately 50 mL with 1% m/v L-cysteine (Sigma, UK) in 5% HCl v/v. Further dilutions in 1% m/v L-cysteine 5% v/v HCl were made up if the stock was expected to result in a concentration outside the calibration range.

Gold concentration was measured on an Agilent 7900 series (Agilent, UK) which was externally calibrated between 0.1 µg/L and 1000 µg/L prepared in 1% m/v L-cysteine 5% HCl v/v from a 1000 mg/L stock in 20% HCl v/v (Alfa Aesar, US). The samples were bracketed by replicates of the lowest and highest concentrations in the calibration curve, and 10 µg/L drift and 10 µg/L calibration check solutions were measured every 5 samples for quality control. Between samples, the system was washed by alternating between solutions containing both L-cysteine (1% w/v) and HCl (5% v/v) or HCl (5% v/v) alone, to reduce the Au memory effect which can otherwise lead to anomalously high signal from low concentration samples. An internal standard (193Ir, 250 µg/L) was mixed online with the samples. ICP-MS analysis was carried out using the following instrument settings: sample uptake rate, 0.3 rps; RF power, 1550 W; Helium collision cell gas flow, 5 L/min; Argon nebulizer gas flow, 1.2 L/min; spray chamber temperature, 2 °C; and integration time, 0.3 s for Au and 0.1 s for Ir. Nanoparticle stocks were subsampled at least 3 times for analysis and the absolute mass of gold was determined as mean ± SD.

Section 1.2: photothermal conversion efficiency equation

Roper *et al.* derived Equation S1 for photothermal conversion efficiency (η) in their 2007 paper on the heat transfer of plasmonically heated gold nanoparticles.[1]

$$\eta = \frac{hA(T_{max}-T_{amb})-Q_0}{I(1-10^{-A_\lambda})}$$ Equation S1

where A is the surface area of the sample cell, Q_0 (J/s) is the baseline power input into a blank quartz cuvette (S2), A_λ is the absorbance of the nanoparticle colloid and h is the heat transfer coefficient defined by (S3).

$$Q_0 = hA(T_{\max\_blank} - T_{amb})$$ Equation S2

$h = \frac{\sum_i m_i C_{p,i}}{\tau A}$ Equation S3

m and C are the mass and heat capacity of each component of the sample cell, which we simplify to the mass and heat capacity of water in the system because of the extremly low relative concentration of gold for each nanoparticle colloid. Combining (S1), (S2) and (S3) we arrive at Equation S4 as presented in the paper. We choose to use extinction (ε_λ) instead of absorbance (A_λ) in (S4) because the nanoparticles we study in this paper have significant scattering properties.

$\eta = \frac{mC(T_{max} - T_{amb}) - Q_{\text{blank}}}{\tau I(1 - 10^{-\varepsilon_\lambda})}$ Equation S4

Where Q_{blank} is calculated from (S5) by irradiating a cuvette containing only water until a temperature plateau is reached.

$Q_{\text{blank}} = mC(T_{\max\_blank} - T_{amb})$ Equation S5

Section 2: Figures and Tables

Table S1. Mass concentration of Au in nanoparticle colloids diluted to ε_{808}=0.1 and measured with ICP-MS across 3 technical replicates.

-

	Mass concentration of Au (µg/ml)
AuNShell	3.15 ± 0.08
AuNR	2.17 ± 0.40
AuNStar	4.04 ± 0.09

Table S2. Time (seconds) required for a voxel containing 0.005 mg/g of AuNRs to reach a hyperthermic temperature of 43°C at different depths within breast tissue at a 0 mm offset from the laser path. The time to reach 43°C was calculated from the simulated power reaching voxels at different tissue depths in the Monte Carlo model, the experimentally determined photothermal mass conversion efficiency (η_m) of AuNRs and Equation 6.

		Tissue depth			
		0 mm (Surface)	10 mm	20 mm	30 mm
Power density	1 W/cm^2	12.0	67.2	600.0	3529.4
	3 W/cm^2	3.6	22.8	200.0	1058.9
	5 W/cm^2	2.4	13.9	120.0	705.8

Table S3. Time (seconds) required for a voxel containing 0.02 mg/g of AuNRs to reach a hyperthermic temperature of 43 °C at different depths within breast tissue at a 0 mm offset from the laser path. The time to reach 43°C was calculated from the simulated power reaching voxels at different tissue depths in the Monte Carlo model, the experimentally determined photothermal mass conversion efficiency (η_m) of AuNRs and Equation 6.

		Tissue depth			
		0 mm (Surface)	10 mm	20 mm	30 mm
Power density	1 W/cm^2	3.0	16.7	150.0	882.4
	3 W/cm^2	1.0	5.8	50.0	294.1
	5 W/cm^2	0.6	3.5	30.0	176.5

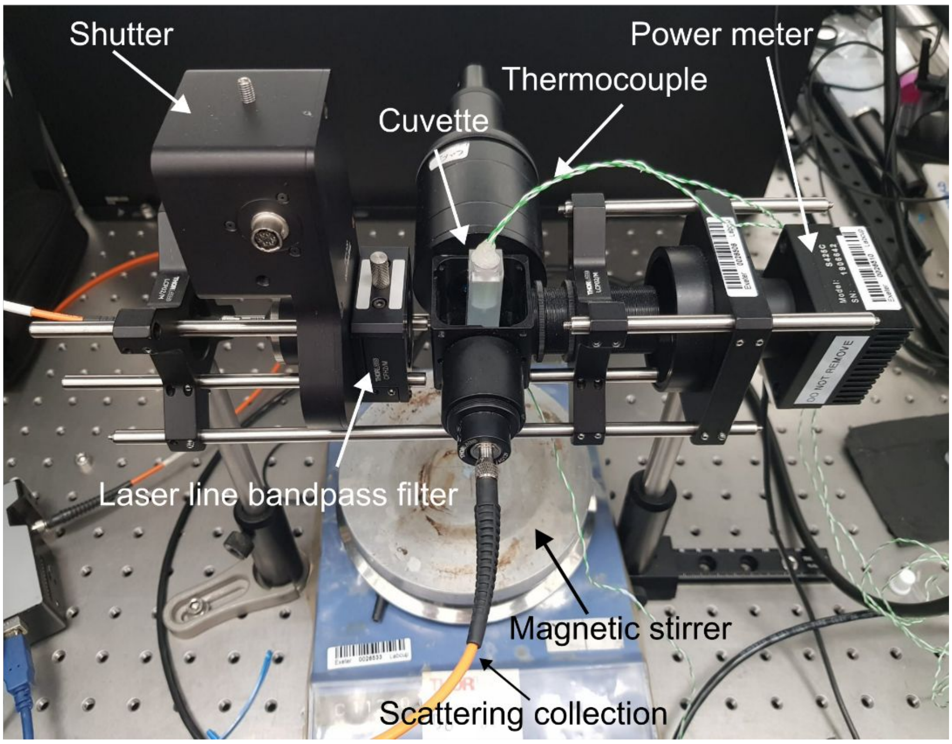

Figure S1. Experimental set-up for PTT experiments.

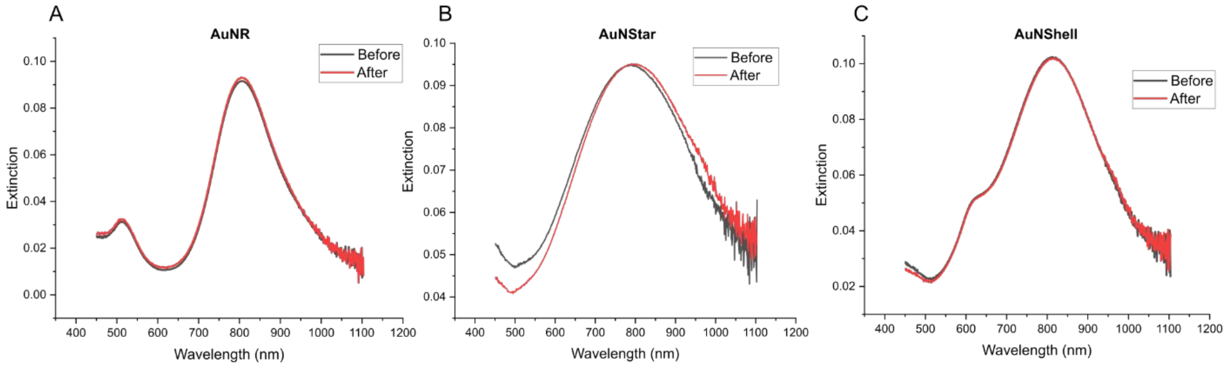

Figure S2. UV-Vis spectra collected before and after irradiating samples for 30 minutes with 1 W of laser power. (A) AuNR, (B) AuNStar and (C) AuNShell UV-Vis spectra showed good stability during laser irradiance with little to no aggregation.

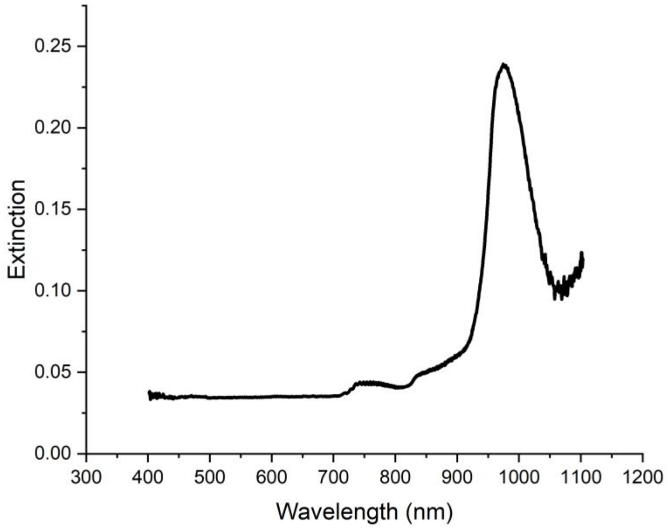

Figure S3. UV-Vis spectrum of the blank quartz cuvette containing water. The small extinction value at 808 is the sum of the light scattered and absorbed by the blank cuvette. The energy absorbed by the cuvette and water is captured in the blank heating curve in Figure 2C and accounted for in calculations of η in the Q_{blank} term. Note that for this spectrum, the background measurement was taken with the sample holder empty (i.e., air).

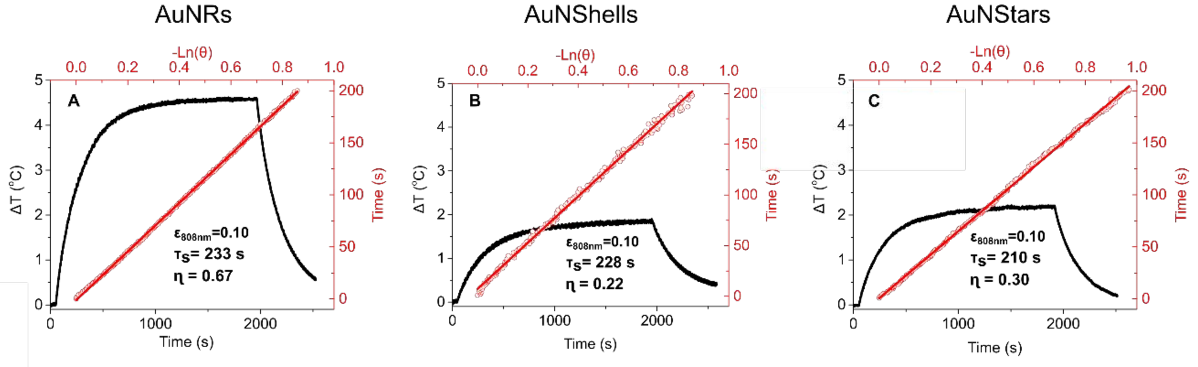

Figure S4. Representative heating curves used to calculate photothermal conversion efficiency (η) for AuNRs, AuNShells and AuNStars. Plots show solution temperature increase above ambient temperature and cooling data used to calculate the time constant τ_s. Solution temperature was measured with thermocouples in cuvette and ambient temperature was measured with a thermocouple attached to the underside of the enclosure. The time constant τ_s was calculated from the gradient of -Ln(θ) vs time (s) according to Equation 4, where $\theta = \frac{T_{amb} - T}{T_{amb} - T_{max}}$.

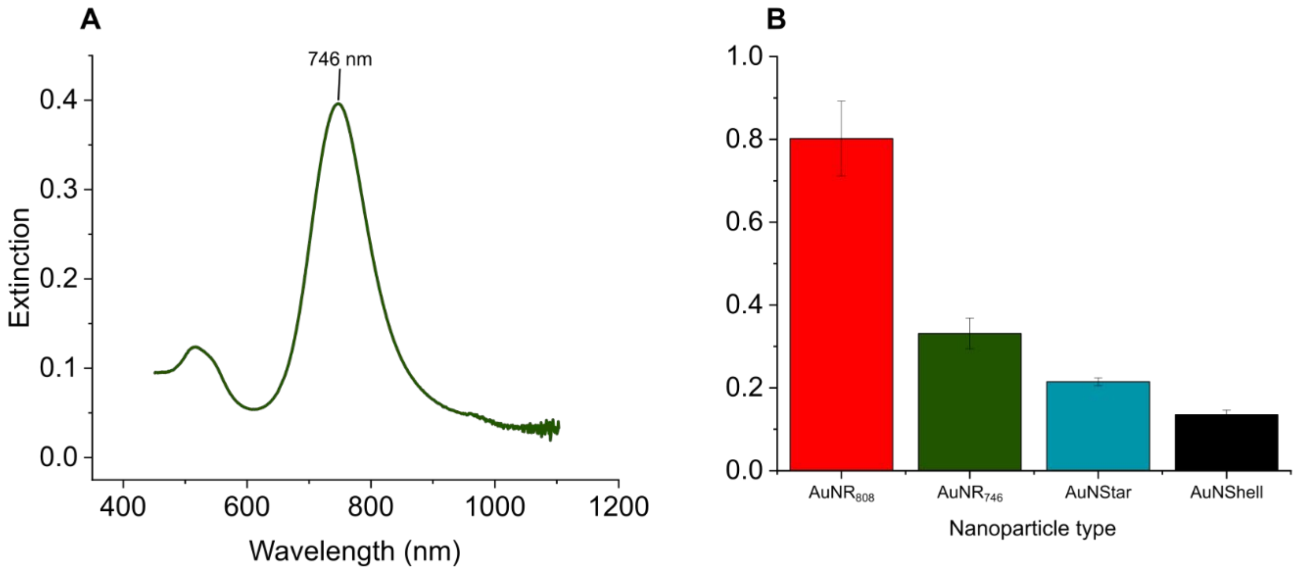

Figure S5. Photothermal mass conversion efficiency (η_m) of off-resonance gold nanorods ($AuNR_{746}$) and comparison with on-resonance nanoparticles ($AuNR_{808}$, AuNShell and AuNStar). (A) UV-Vis extinction spectrum of $AuNR_{746}$. We synthesized these AuNRs using Yang *et al*.'s method[2] with ascorbic acid volume adjusted to 63 µl to get an aspect ratio of 3.37 ± 0.2 (length = 46.4 nm, width = 13.9 nm, by TEM) and plasmon at 746 nm. (B) $\eta_m$for each nanoparticle type. Off-resonance $AuNR_{746}$ show lower η_m than $AuNR_{808}$ because a higher mass concentration of nanoparticles is required to absorb an equivalent amount of light at 808 nm. However, the high photothermal conversion efficiency of nanorods [3] means that η_m for $AuNR_{746}$ remains higher than for AuNstar and AuNShell.

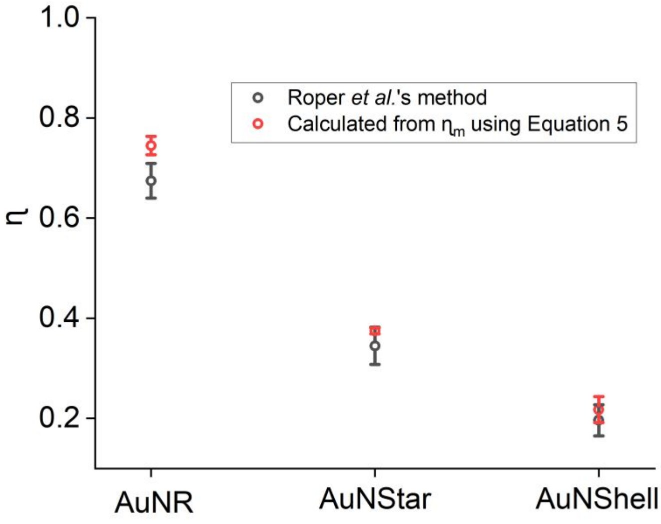

Figure S6. A comparison of photothermal conversion efficiency (η) calculated using Roper *et al*.'s method (Equation 3, heating and cooling curves in Figure S2, laser power = 1 W and combined heating and cooling time of 40 minutes) and calculated from η_m (Equation 5, heating data in Figure 3A, laser power = 500 mW, total heating time of 60 seconds).

References

(1) Roper, D. K.; Ahn, W.; Hoepfner, M. Microscale Heat Transfer Transduced by Surface Plasmon Resonant Gold Nanoparticles. *J. Phys. Chem. C* **2007**, *111* (9), 3636–3641. https://doi.org/10.1021/jp064341w.

(2) Yang, J. A.; Lohse, S. E.; Boulos, S. P.; Murphy, C. J. The Early Life of Gold Nanorods: Temporal Separation of Anisotropic and Isotropic Growth Modes. *Journal of Cluster Science* **2012**, *23* (3), 799–809. https://doi.org/10.1007/s10876-012-0474-y.
